# Sustainable Agriculture through Multidisciplinary Seed Nanopriming: Prospects of Opportunities and Challenges

**DOI:** 10.3390/cells10092428

**Published:** 2021-09-15

**Authors:** Amruta Shelar, Ajay Vikram Singh, Romi Singh Maharjan, Peter Laux, Andreas Luch, Donato Gemmati, Veronica Tisato, Shubham Pratap Singh, Maria Fernanda Santilli, Akanksha Shelar, Manohar Chaskar, Rajendra Patil

**Affiliations:** 1 Department of Technology, Savitribai Phule Pune University, Pune 411007, India; amrutavijaykumarshelar@gmail.com; 2 Department of Chemical and Product Safety, German Federal Institute for Risk Assessment (BfR), Max-Dohrn-Strasse 8-10, 10589 Berlin, Germany; Romi-Singh.Maharjan@bfr.bund.de (R.S.M.); Peter.Laux@bfr.bund.de (P.L.); Andreas.Luch@bfr.bund.de (A.L.); 3 Department of Translational Medicine, University of Ferrara, 44121 Ferrara, Italy; cet@unife.it (D.G.); veronica.tisato@unife.it (V.T.); 4 Faculty of Informatics, Otto von Guericke University, 39106 Magdeburg, Germany; shubhamp.singh@yahoo.com; 5 The Nanoinformatics Innovation Centre, La Plata, Buenos Aires C1062, Argentina; mfsantilli@gmail.com; 6 Department of Microbiology, Savitribai Phule Pune University, Pune 411007, India; akankshavijaykumar2@gmail.com; 7 Ramkrishna More Arts, Commerce and Science College, Pune 411044, India; dean.st@unipune.ac.in; 8 Department of Biotechnology, Savitribai Phule Pune University, Pune 411007, India

**Keywords:** seed priming, nanotechnology, germination, seed resistance, sustainability, cold plasma technology, machine learning

## Abstract

The global community decided in 2015 to improve people’s lives by 2030 by setting 17 global goals for sustainable development. The second goal of this community was to end hunger. Plant seeds are an essential input in agriculture; however, during their developmental stages, seeds can be negatively affected by environmental stresses, which can adversely affect seed vigor, seedling establishment, and crop production. Seeds resistant to high salinity, droughts and climate change can result in higher crop yield. The major findings suggested in this review refer nanopriming as an emerging seed technology towards sustainable food amid growing demand with the increasing world population. This novel growing technology could influence the crop yield and ensure the quality and safety of seeds, in a sustainable way. When nanoprimed seeds are germinated, they undergo a series of synergistic events as a result of enhanced metabolism: modulating biochemical signaling pathways, trigger hormone secretion, reduce reactive oxygen species leading to improved disease resistance. In addition to providing an overview of the challenges and limitations of seed nanopriming technology, this review also describes some of the emerging nano-seed priming methods for sustainable agriculture, and other technological developments using cold plasma technology and machine learning.

## 1. Introduction

**Figure 1:**
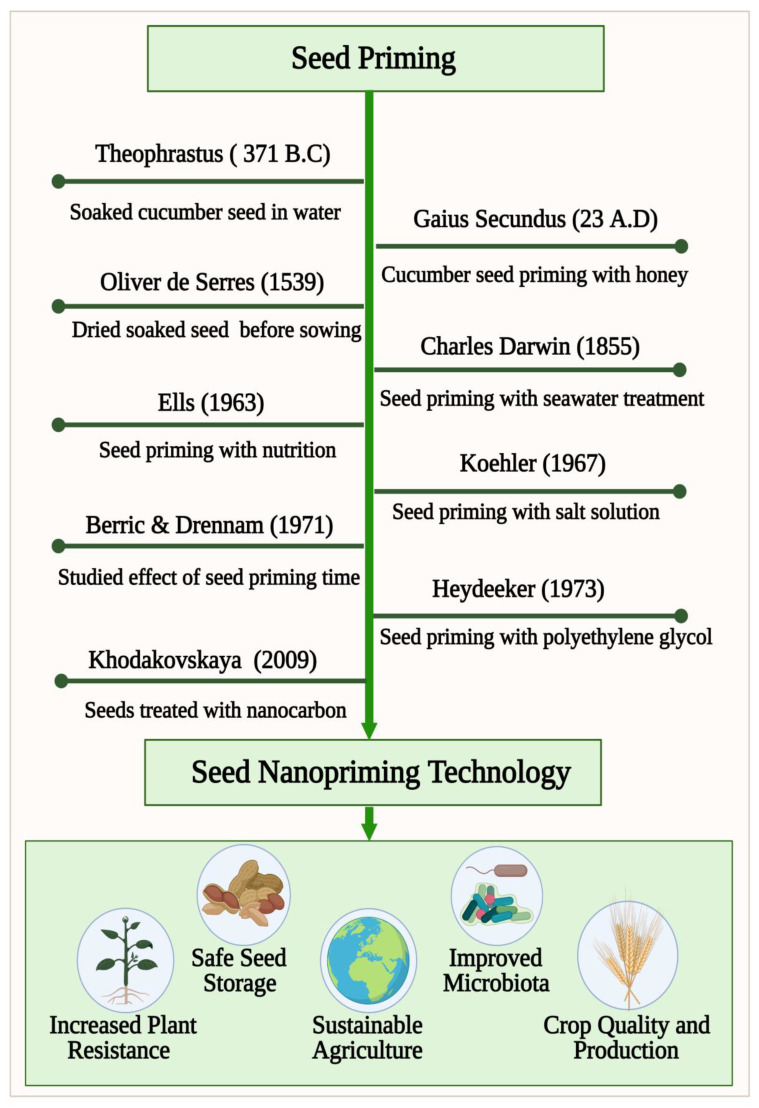
A timeline of seed priming development towards nanopriming technology to improve seed germination. For sustainable food production, nanopriming improves the ability to withstand environmental stressors, improves seed storage, and boosts microbiota.

World food production must increase 50% by 2050 to meet the needs of 9 billion people [1]. The growing food demand and rapidly changing climatic conditions across the world motivates us to look for technological solutions that can provide food security for the future generations. Seed is a primary requirement for crop production, which carries the genetic potential of the variations and determines the ultimate productivity. Therefore, seed production is always the basic pre-requisite of any food security undertaking. A resilient and climate hardened seed can give the maximum output to the farmers. To increase the seed vigor and crop production, different chemical-based fertilizers and pesticides are used extensively in agriculture. In light of leaching, degradation, hydrolysis, and pollution associated with conventional chemical-based practices, they are being discouraged [2].There is an urgent need to develop a sustainable technology that can contribute to the green revolution to address these growing concerns and to restore the damage caused to the ecosystem [3]. Seed priming is an innovative sustainable seed technology to increase the seed vigor and crop production without harming the ecosystem [4,5,6,7]. As mentioned in Figure 1, priming of seeds dates back to Theophrastus, (371–287 B.C.) who, observed during an investigation that soaking cucumber seeds in water causes their germination to be faster and more uniform than unprimed seeds (enquiry into plants, book VII, I.6). A similar preparation of cucumber seeds in honey and water for seed germination was reported by the Roman naturalist Gaius Plinius Secundus (23–79 A.D.) in Gaius’ Encyclopedia (Gaius 1949–1954). The French botanist Oliver de Serres, in 1539–1619, found that soaking seeds in manure water for 2 days and drying them prior to sowing was an effective cure for poor crop growth. The osmo-priming process was tested on lettuce and cress seeds in seawater by Charles Darwin, who observed that the primed seeds germination was higher than that of non-primed seeds. By identifying the critical parameters of seed treatment, Ells (1963) presented the modern concept of seed priming. His experiments with nutrient solutions showed a high germination rate. Khodakovskaya et al. published one of the first studies to demonstrate the potential for nanomaterials to affect seed germination [8]. The latest innovative technique with potential application in seed priming is seed nanopriming, an important emerging seed technology that blends seed priming science with updated nanotechnology [9,10]. A lot of attention has recently been brought to the development and optimization of nanomaterials for application in agriculture, including improved growth, plant protection, and overall performance. Agricultural nanomaterials are still in a juvenile state in terms of their application to sustainable agriculture [11]. Nanomaterials will be applied to multiple functions in agriculture as the understanding of nanotechnology increases. It has been noticed that most agricultural lands are affected by abiotic stress factors such as salinity and drought, which is limiting plant distribution in the habitat [12]. In order to combat abiotic stress factors, engineered nanoparticles have been found to be a promising alternative [13]. Recent studies have shown that nanomaterials can significantly impact plant metabolism, genes expression, and antioxidant enzyme activity [14]. Abiotic stress can be improved by nanoparticles such as halloysite, cerium oxide, chitosan-selenium and titanium dioxide by enabling their antioxidant system to perform better [15,16,17,18]. The use of nanoparticles as seed priming agents has demonstrated encouraging results in the field of crop productivity and seed germination [19]. By using nanotechnology, it is possible to release priming agents at specific sites and in controlled ways, which is revolutionary for ecosystems, crop improvement, animal health, and pesticide use. Nanopriming is an ongoing effort to create nano-agrochemicals for releasing specific nutrients in a controlled manner, which maintains soil fertility. Using high quality seed detection technologies makes it more likely to identify a variety of better crops since seed quality is an important factor in crop production. An increasing number of effective methods are needed that can detect nanoprimed seed quality in a non-destructive, objective manner as quickly as possible. The presented review describes the potential application of engineered nanomaterials in seed nanopriming for sustainable agriculture and machine learning technology for detection and classification of nanoprimed seeds to improve crop production.

## 2. Seed Nanopriming Technology

**Figure 2:**
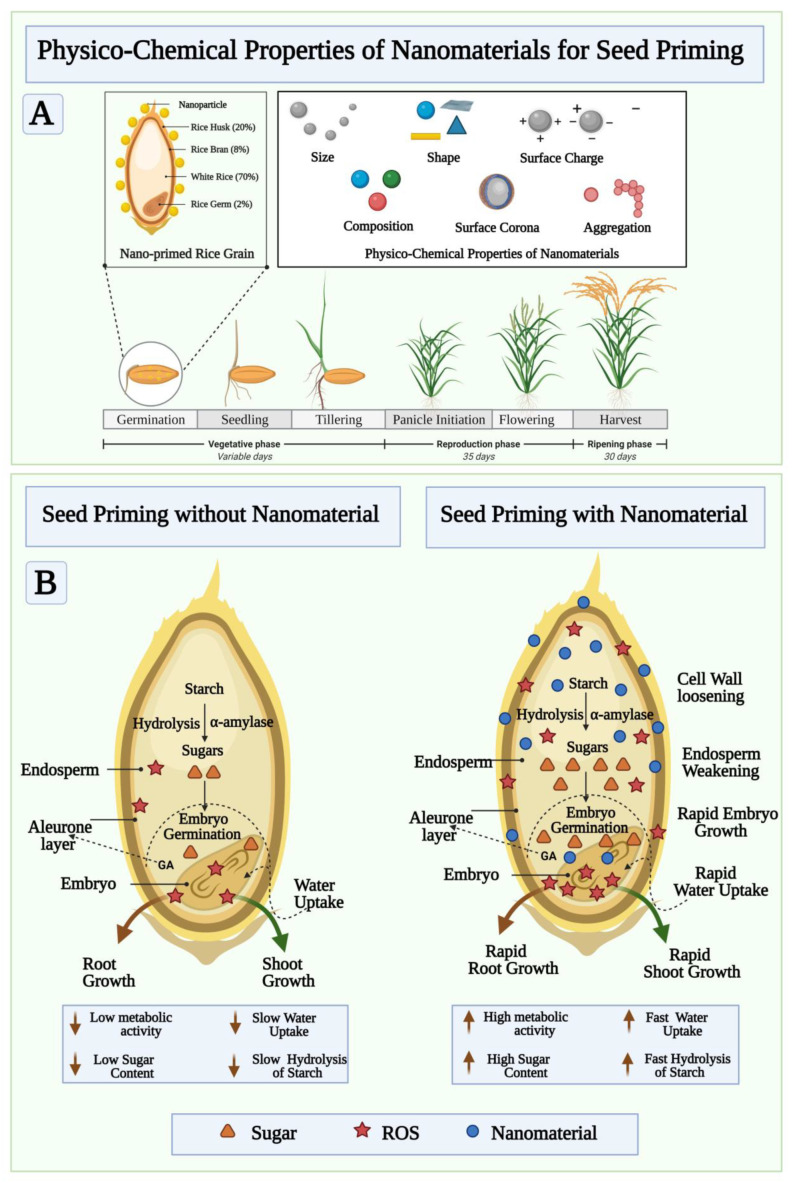
Physicochemical properties of nanomaterials influencing seed priming and its proposed mechanism. (**A**) The nanomaterial’s size, shape, and surface chemistry make them a versatile candidate to modulate the seed priming phenomena via size-mediated diffusions and relocating to specific regions of the seed to activate synergistic mechanisms resulting in germination. (**B**) The versatile catalytic function of NMs selectively play role in promoting enzymatic and biochemical pathways to promote root/shoot growth.

Seeds are miniature plants which, when fertilized, hatch into ovules containing both a germinating embryo and enough food to grow. A variety of treatments can be applied to a seed before planting in order to enhance its quality and potential yield. The process of priming seeds, in its broadest sense, involves soaking them in a solution where enough water is absorbed so that germination of the seed becomes possible, but not enough to allow the radicle to protrude through the seed coat [20]. Using this technique, seeds are advanced to an equal stage of germination to facilitate emergence from seed quickly and uniformly [21]. Thus, priming of seeds is a very important practice in order to ensure seed productivity [22]. Seed priming methods include hydro-priming, halo-priming, osmo-priming, hormonal priming, solid matrix priming, and bio-priming to stimulate seeds, encourage germination, and reduce environmental stress. Among the most well-known and cost-effective pre-sowing seed priming methods, hydro-priming involves treating seeds with water before sowing. The seeds are soaked in water, then re-dried to their original moisture content [23]. The process of halo priming involves treating seeds with inorganic solutions such as sodium chloride, potassium nitrate, calcium chloride, and calcium sulphate to improve germination. A known application of halopriming during the germination, seedling emergence, emergence of plants, and growth of plants is well known [24]. In osmo-priming, seeds are soaked in different concentrations of osmotic solutions. Different osmotic solutions are used depending on the species, including sugar, polyethylene glycol, glycerol, sorbitol, and mannitol and then air drying is followed by sowing. Solutions containing low water potential facilitate seed imbibitions and, therefore, early stages of germination occur [25]. Hormonal-priming involves the use of hormone solutions to prime seeds. During hormonal priming, plant growth regulators are used to imbibe seeds that have direct effects on seed metabolism. Hormo-priming uses various regulators such as abscisic acid, salicylic acid, ascorbic acid, cytokinins, auxins, gibberellins, kinetin, ethylene, and polyamines [26]. In solid matrix priming (SMP or Matri-conditioning), seeds are mixed in known proportions with a solid material and water. Water-absorbing seeds will reach a point of equilibrium, precisely at which priming will take place. A thorough washing and drying follow the separated seeds from the matrix. A hydrated, metabolically active seed can then be achieved, which is an important germination step [27]. As an ecological approach, bio-priming involves seed inoculation with beneficial organisms to protect seed from disease. Seed treatment with this new trend involves hydrating seeds with beneficial microorganisms and improving germination procedures. In terms of disease management, biopriming provides better results than pelleting or coating [28]. Nanopriming, a technique that involves seeds soaking in nanomaterials, has been shown to facilitate germination and growth by allowing nanoparticles to penetrate the seed coat and increase water uptake. When compared with unprimed seeds or seeds treated with other priming agents, nanopriming improves storage, quality, seedling emergence, yields, and tolerance to environmental stress [7]. In addition, nanopriming of seeds can help prevent diseases caused by pathogens present in seeds. A high percentage of nanomaterials are retained on the seed surface as coatings when nanoparticles are absorbed, and a tortuous pathway prevents uncontrolled water uptake while reducing gas permeability, which leads to more stable seed storage. A nanopriming process can alter the metabolism of seeds and signalling pathways, thereby influencing their germination and growth. In addition to affecting almost every existing scientific field, nanoparticles can gain significant impact on agricultural sustainability by being introduced to the agricultural domain [7]. Studies have demonstrated that nanoparticle application can stimulate germination and growth of plants in numerous ways. Nanoparticles are effective because of their small size and unique physio-chemical properties, which make them an ideal seed priming agent [21]. Nanoparticles are molecular or atomic aggregates that have a measured dimension between 1 nm and 100 nm and which may produce significantly higher chemical and physical properties than typical bulk materials [29]. It is important to note that nanomaterials have a wide range of physio-chemical properties depending on the shape, size, surface area, surface/volume ratio, chemical behavior, particle charge, production method, coating, and so forth (as shown in Figure 2A). The unique features of nanomaterials, such as their high surface to mass ratio, enables them to enhance catalysis and deliver materials of interest, as well as adsorb substances of interest. The nanopriming process triggers a special metabolic reaction that is naturally triggered during the early stages of germination, as shown in Figure 2B. It increases seed germination, by modulating the metabolism of seed, which leads to enhanced water uptake, starch hydrolysis rate, cell wall loosening, endosperm weakening, rapid embryo growth, and rapid root-shoot development. The nanopriming method improves the emergence of seedlings, their growth, production, and quality [30]. The cellular and molecular mechanisms of plant interaction with the environment are also modulated by seed nanopriming. The goal of using nanoparticles in agriculture and natural ecosystems is to increase the performance and sustainability of plants and soil by using less of the input parameters defined above [31,32]. It is important to include different factors that affect priming success, such as the amount of water in the soil, the kind of priming treatment, the amount of time the seeds are exposed to the treatment, and the conditions in which the seeds are stored. Numerous studies have demonstrated the ability of nanoparticles to penetrate seed coats and enhance water uptake over time, which facilitates germination and flowering [33,34]. As seed pretreatment agents, several metal-metal oxide nanoparticles and carbon-based nanoparticles have been applied to enhance germination and seedling growth of some crops and to strengthen their stress tolerance [35,36,37]. Seed priming, in which seeds are re-dried to their original moisture content before planting, has been used in only a few studies, but it is not widely used. In this case, seed nanopriming would provide a different mechanism to that of pre-sowing seed treatment without drying. Additionally, comprehensive studies have not been conducted on the physiological and molecular mechanisms of nanopriming on seed germination, thus there are many unanswered questions, particularly concerning the mechanism behind nanoparticles-induced seed germination. A summary of the types of nanoparticles that are used for seed priming is shown in Table 1, with their potential effects as stimulants or protective against biotic and abiotic stress.

## 3. Molecular Targets to Seed Nanopriming

An analysis of the studies shows that the benefits of nanomaterial in promoting seed germination include (a) developing nanopores in the seed coat, (b) introducing reactive oxygen species (ROS) to the seed, and (c) using the nanocatalyst to boost enzyme activity at the starch-degrading site (Schematic Figure 2B) [35]. Although the exact mechanism behind seed nanopriming is not clearly understood, it appears that nanoparticles can induce physiological effects on seed germination. A nanomaterial that penetrates the seed coat and creates small pores can lead to increased water uptake and upregulation of aquaporin gene expression. Nano-pretreated seeds accumulate more ROS during germination than unprimed seed and other priming treatments, suggesting that significant ROS is needed to stimulate seed germination as shown schematically in Figure 2B. A study by Kibinza et al. demonstrated that osmo-priming sunflower seeds increases expression of a gene encoding catalase, suggesting that catalase may be a key enzyme responsible for the recovery of vigor in older seeds [56]. The enzyme superoxide dismutase scavenges O_2_•− and converts it into H_2_O_2_, and the enzyme catalase transforms H_2_O_2_ into water. A significant increase in antioxidant enzyme amounts could be the result of nanoparticle-mediated reduction of ROS. Due to the relationship between reactive oxygen species and antioxidant enzymes, seeds that are nanoprimed tend to contain a lot of antioxidant enzymes through nanoparticle-mediated ROS mitigation. Furthermore, aquaporin and ROS-mediated interactions have been found to influence seed development [57]. The activation of seed germination could be achieved through aquaporins and ROS. Several studies suggest that aquaporins facilitate the transfer of H_2_O_2_ and ROS through biological membranes as well as water uptake [58]. As a result of the nanopriming process, nanopores are formed, allowing rapid influx of water into the seeds, and aquaporin genes are activated. For seed growth and germination to occur, seed antioxidant systems must regulate ROS in order to trigger oxidative signaling molecules to function. The nanopriming process leads to increased soluble sugar levels. Sugar concentrations in the cells can reduce osmotic potential, which reduces the water potential. In this way, the difference (gradient) between the water potential outside and inside the tissues increases, facilitating water movement into the seeds. Nanoprimed seeds have increased soluble sugars content, resulting in the increase of amylase activity, which leads to an increase in water uptake in the seeds due to the change in internal osmotic potential due to the increase of soluble sugars (solutes). Studies have demonstrated that ROS, including OH, contribute to radiation growth, cellular reorganization, and endosperm and testicular wasting [59]. Nanoparticles can generate ROS by triggering the formation of •OH. A theory suggests that during the short period of time (i.e., 24 h) spent soaking seeds in nanopriming solutions, the OH produced by bound nanoparticles would facilitate a process of loosened cell walls, which would stimulate the growth of seedlings [60]. As a nanocatalyst, nanoparticles absorbed from the seeds may be crucial in accelerating the reaction rate of starch hydrolysis catalyzed by α-amylase. (Figure 2B) [35]. A recent study showed that nanoparticles can act as nanocatalysts, and that soluble starch can be rapidly digested with nanosilver [61]. As reported by Mahakham et al. [35] the aquaporin gene (PIP1;1 and PIP2;1) expressed higher levels in rice seeds primed with silver nanoparticles. In conjunction with triggering the expression of aquaporin genes, aquaporins (water channels) are trans membrane proteins that facilitate the movement of water and metabolic gases across biological membranes, and regulate water homeostasis. Nanopriming of rice seeds is an efficient method for boosting their starch metabolism. The hydrolytic enzyme α-amylase converts reserved carbohydrates into soluble sugars during seed germination, which keeps the respiration metabolism active until sufficient photosynthesis occurs. A study conducted by Jing et al. [62] examined the transcriptomes of cotton seeds treated with poly(acrylic acid)-coated cerium oxide nanoparticles (PNCs) and then exposed to saline stress. Compared to unexposed control groups, ROS are accumulated under conditions of salinity stress. When salinity stress is applied to nanoprimed seed, the expression of 13 genes related to ROS signaling pathways and 10 genes related to ion homeostasis was detected. Plants produce antioxidant enzymes to counteract oxidative damage, including peroxidases (POD), glutathione S-transferases (GST), and peroxiredoxins (PRX). Comparing seedlings under salt stress, POD and GST were significantly up regulated, while PRX was down regulated by PNC priming. A PNC priming also increases magnesium levels in cotton roots, as well as genes (CAD1 and TPS) with functions related to terpene synthase. In response to salt stress, PNC seed priming regulates cotton seedling development through signaling ion and antioxidant pathways. Among the oomycete species investigated in the study, Siddaiah et al. [63] found that chitosan nanoparticles showed antimicrobial activity against *Sclerospora graminicola*, which causes downy mildew. By priming seeds with chitosan nanoparticles, plants display improved immunity, with increased gene expression of alkaline peroxidase, polyphenoloxidase, and phenylalanine ammonia lyase. The nanochitosan seed priming over expression involves PR1 and PR5, which are involved in the salicylic acid pathways. Using zinc oxide nanoparticles to combat environmental stress factors was studied by Chaudhary et al. [38]. In response to ZnO nanopriming, miR156 and miR159 expression increased, which plays a significant role in helping plants resist biotic and abiotic stresses. A study by Ye et al. [50] demonstrated that manganese nanoparticles improved seedling growth under salinity conditions. Nanopriming with manganese upregulated SOD (superoxide dismutase), which offers protection against ROS damage and prevents phytotoxicity. It is possible that nanoparticles are present within the seed to account for this behavior. Nanoparticles appear to enhance starch hydrolysis, but the exact mechanism is still unclear [64]. Nevertheless, further research is warranted to determine the exact mechanism.

## 4. Effects of Seed Nanopriming under Abiotic and Biotic Stresses

**Figure 3:**
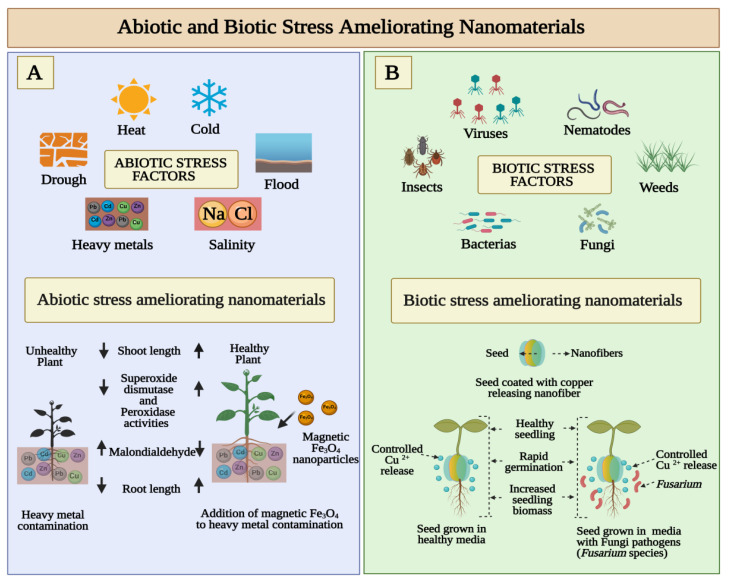
Effect of nanopriming agents to improve seed germination in abiotic and biotic stress conditions. (**A**) Environmental conditions in combination with soil quality may pose stress to different phases of seed germination affecting biochemical pathways involved in the root-shoot development. The metal chelators and biocatalytic NPs could play a rescue role mitigating those abiotic conditions, giving a healthy plant development. (**B**) Seed nanopriming with broad spectrum NMs with known microbicidal and anti-parasitic effect improve seed germination in biotic stress conditions boosting overall crop yield.

Abiotic and biotic stresses cause damage to seed growth and eventually lead to economic losses. In the wake of global warming and climate change, seeds are subject to an increased number of biotic and abiotic stress combinations, which negatively affect their growth and yield [65]. The simultaneous presence of abiotic stress factors such as drought, flood, salinity, heavy mineral contamination, cold and heat has been shown to severely deter seed germination (Figure 3A). The biotic stress that is exhibited in Figure 3B includes a variety of plant pathogens including bacteria, fungi, viruses, nematodes, insects, and others. As a result of pathogen infection, changes in plant physiology often result in reduced biomass, early flowering, decreased seed set, accumulation of protective metabolites, and many other changes [66]. Researchers have reported that a variety of nanomaterials reduce biotic and abiotic stress and improve seed germination [67,68].

### 4.1. Abiotic Stress Ameliorating Nanomaterials

By seed priming with cerium oxide nanoparticles, An et al. studied the molecular mechanisms behind plant salinity stress tolerance. Poly (acrylic acid)-coated cerium oxide nanoparticles (PNC) show morphological, physiological, biochemical, and transcriptomic effects on cotton seedling priming under salinity stress, according to An et al. An advantage of PNC nanoparticle seed priming is that it can be used to increase a crop tolerance to stress during the early seedling stage in a sustainable, practical, and scalable way. These results showed promising molecular mechanisms that could be synergistically operated to enhance plant salt tolerance [62]. According to a recent study published by Baz et al., nanopriming with water-soluble carbon nanoparticles (CNPs) significantly increases seed vigor and seedling growth of lettuce under salinity stress. Under high temperatures and salinity stress, CNPs have been shown to significantly promote seed germination. A CNP-assisted nanopriming treatment enhanced lateral root growth but slightly inhibited the elongation of primary roots, resulting in a balanced accumulation of chlorophyll in high salinity stress [69]. The effects of manganese (III) oxide nanoparticles (MnNPs) on the salinity stress of *capsicum annum L* were studied by Ye et al. It showed that MnNPs can penetrate seed coats and form corona complexes. The results of the study demonstrate that MnNPs can modulate biochemical interactions in seeds that exhibit salinity tolerance in order to promote sustainable agriculture [50]. The study showed that nano-silica primed zea mays seeds had a higher rate of germination and a higher seedling vigor index. The priming process helps to increase the activity of antioxidant enzymes, which can suppress lipid peroxidation by suppressing the production of ROS under salinity stress. Additionally, priming reduces abscisic acid content while increasing gibberellin content. As a result of this hormonal balance, the hydrolysis enzymes (amylase and lipase) are activated. It was found that nano-silica priming enhanced the metabolic activity of maize seeds when exposed to salinity [70]. Khan et al. investigated the effect of nano ZnO and nano Fe on cadmium accumulation in wheat. Nanoprimed seeds show increased length of spikes, shoots, and roots, as well as increased grain size. Superoxide dismutase activity and electrolyte leakage were reduced in nanoprimed seeds. Nanoparticles in seeds increase wheat biomass and nutrient content as well as decreasing Cd toxicity overall [71]. The role of bulk and nanosized SiO_2_ particles in fenugreek germination has been investigated by R. Ivani et al. A study concluded nanosized particles of SiO_2_ protect fenugreek seeds from salt stress and improve growth attributes [72]. A study by S. Hojjat et al. examined the effects of silver nanoparticles on the germination of fenugreek seeds under salinity conditions. AgNPs improved salinity tolerance in fenugreek seedlings. This may increase various plant defense mechanisms that reduce salt stress [73]. In a study by Konate et al., magnetic (Fe_3_O_4_) nanoparticles were shown to mitigate heavy metals uptake and toxicity in wheat seedlings. Physiological mechanisms were investigated in wheat seedlings to determine how magnetic nanoparticles (nano-Fe_3_O_4_) mitigated the toxic effects of heavy metals (Pb, Zn, Cd, and Cu) [74].

### 4.2. Biotic Stress Ameliorating Nanomaterials

As a bioprotectant against rice borne pathogens *P. grisea* and *X. oryzae*, Sathayabama and Muthukumar investigated the antimicrobial activity of chitosan guar nanoparticles (CGNP)**.** The treated rice showed no symptoms of blast disease. Rice plants with CGNP-applied antimicrobial protection displayed an enhanced rate of seed germination and growth [75]. T. Xu et al. demonstrated enhanced seedling development and agrochemical delivery by biodegradable, biopolymer-based nanofiber seed coatings. The germination and subsequent growth of nanofiber-coated tomato and lettuce seeds were studied in a greenhouse, with and without a fungal pathogen (*Fusarium* species) as shown in Figure 2B. Based on recent observations, it appears to be possible to use nanomaterials to deliver active ingredients at precise locations. Nanofiber seed coatings provide precise agrochemical delivery and significantly improve seedling germination and biomass in comparison to conventional film coating techniques used in the industry due to their nanofibrous structure and controlled release kinetics [76]. The effects of cinnamaldehyde encapsulated mesoporous silica nanoparticles (MSNP) on seed borne diseases were studied by Bravo Cadena et al. Based on the results of this study, it is clear that MSNP significantly enhanced the antimicrobial activity of plant products, which allows the use of volatile biocides such as essential oils at very low concentrations to prevent microbial diseases in crop plants [77]. Choudhary et al. evaluated the effect of zinc chitosan nanoparticles as a seed priming agent and foliar application to maize plants. An in vitro study showed that seed nanopriming with Zn-chitosan nanoparticles enhanced seed germination and inhibited fungal growth. These results showed that Zn-chitosan nanoparticles have strong fungicidal activity, are an effective micronutrient fortifier, and could stimulate maize crop growth [38].

## 5. Effect of Combined Seed Priming Treatment of Nanomaterials with Microbes

**Figure 4:**
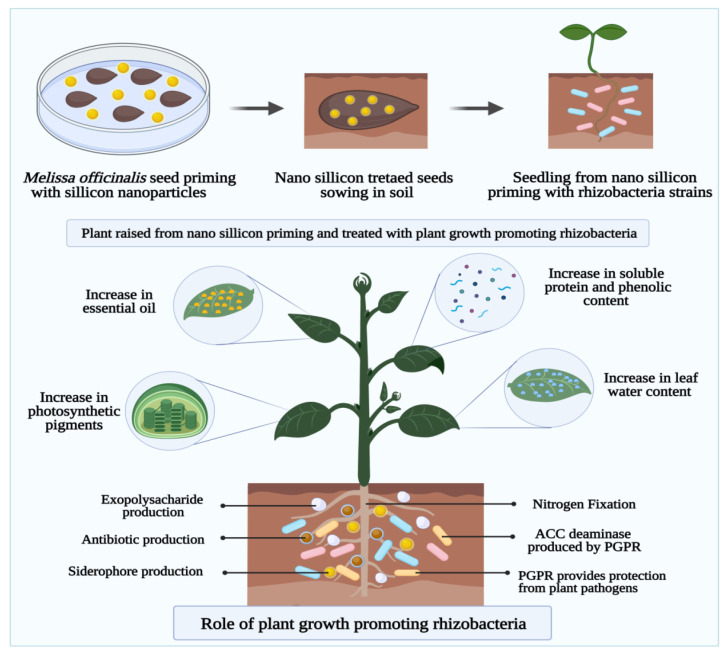
The plant growth-promoting rhizobacteria (PGPR) combined seed nanopriming treatment with a selection of microbicidal nanomaterials without influencing the PGPR spares beneficial microbes of the soil and plant tissues. This promotes N_2_-fixation improves harvest quality and yield via selectively increasing threshold towards biotic stresses.

Seed germination and seedling growth characteristics of several plant species have been enhanced by nanopriming with a variety of nanomaterials [9,44]. Several studies on the interaction between nanomaterials and plant-benefitting bacteria, called Plant Growth-Promoting Rhizobacteria (PGPR), have been identified as a novel approach in sustainable agriculture [78]. Through the application of both PGPR and nanomaterials, plants may be able to resist environmental stress through several physiological and morphological mechanisms, such as improved root system nutrient uptake and induced expression of cellular antioxidative enzymes [79]. By using silicon nanoparticles as a seed priming agent, Mahakham et al. studied the role of silicon nanoparticles in the cell growth process. This study examined the effect of silicon nanoparticle-mediated seed priming on *Pseudomonas* species enhancing growth, physiology, and antioxidant metabolism in *Melissa officinalis* L. According to Figure 4, seed priming, pre-sowing, and seedling inoculation with bio-elicitor improves plant growth and phytochemical constituents. Combined with silicon nanoparticles, *P. fluorescens* and *P. putida* significantly increased plant biomass indices, the relative water content of leaves, photosynthetic pigment values, essential oil yield, and all major components (except thymol). It has been found that seed priming with nano-silicon particles combined with inoculation with pseudomonas strains boosts the number of both primary and secondary metabolites in lemon balm plants [35]. Shcherbakova et al. demonstrated that pre-seed treatments with microbial inoculants and molybdenum (Mo) nanoparticles. The goal of this study was to determine the effects of pre-seed treatment with microbial inoculants and Mo nanoparticles on the composition of root exudates of chickpea plants and the diversity of microbes in the rhizosphere. Shcherbakova et al. examined the activity of enzymes involved in the antioxidant protection system and the formation of plant-microbial systems induced by nodules and rhizosphere bacteria as well as Mo nanoparticles before sowing seeds [80]. De Gregorio et al. investigated the application of beneficial rhizobacteria immobilized in nanofibers to bioinoculants soybean seeds. Their findings showed that inoculating seeds with PGPR provides soil with a significant number of beneficial microorganisms. A nanofiber-immobilized rhizobacteria-coated soybean seed was also evaluated for its influence on bacterial survival during seed storage and on characteristics of germination and plant growth. Seed coating with *P. agglomerans* improved germination, size, and weight of the roots. Moreover, seed coating with *Bacillus californianus* increased leaf number and dry weight. In view of this, the technique applied in the current study to prime seeds with nanofiber-immobilized PGPR could be regarded as a promising eco-friendly strategy to enhance soybean production by using microbial inoculants [81]. According to Taran et al., colloidal solutions of metals for micronutrients enhance plant health by enhancing resistance to exposure to unfavorable environmental conditions and enhancing plant nutrition by increasing the penetration of nanoscale elements in the cell walls. Combining seed treatment with colloidal solution of Mo nanoparticles and microbial preparation results in a four-fold increase in nodule formation compared to control plants [82].

## 6. Effect of Combined Seed Priming Treatment of Nanoparticles and Cold Plasma Technology

**Figure 5:**
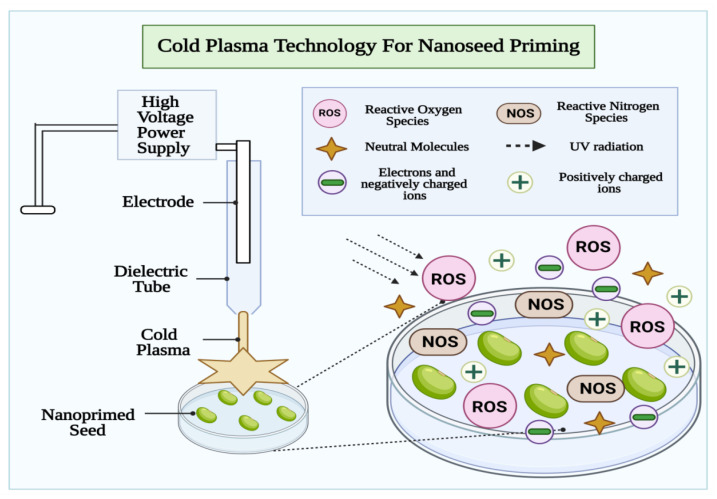
The cold plasma (dielectric barrier discharge) mediated seed priming improve seed vigor via synergistical activation of plant defense machinery against ROS, supporting redox homeostasis in plant. Bases on cold vs. non-thermal plasma used in seed priming, the energetic electrons, charged particle, and reactive species are produced, which dissolve tough seed coat promoting water uptake and drought yield in the crop.

**Figure 6:**
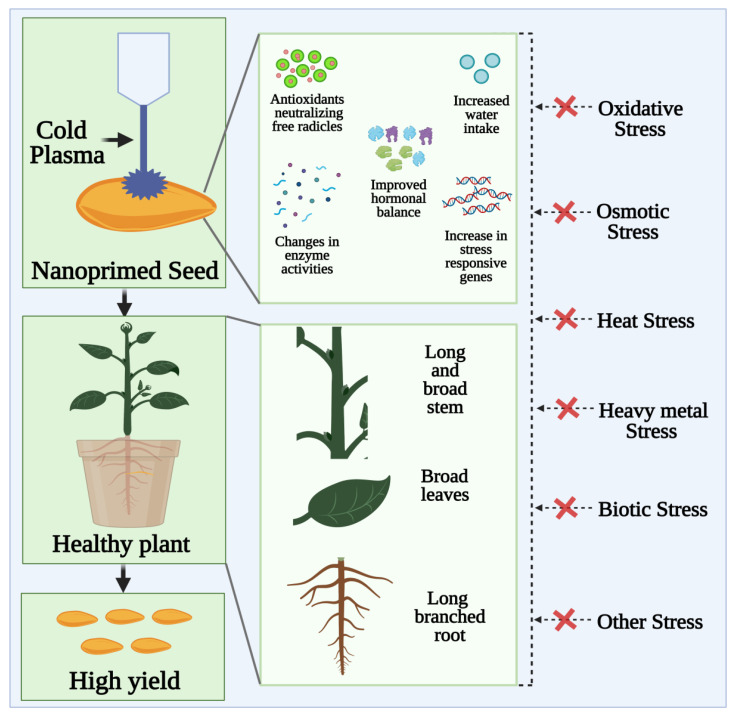
As an alternative to ecotoxic chemical treatments, non-thermal and cold plasma-based seed nanopriming has advantages in managing environmental stressors towards improving seed vigor and enhancing seed germination. The enhanced antioxidant system and activated defense response towards improved physiological processes; seed scarification and pathogen inactivation via plasma affects seed surface environment ameliorating improved features at molecular level and leave/root/shoot proportions.

The combined potential benefits of working with cold plasma and metal-based nanoparticles seed priming are rarely studied. The reactive oxygen and nitrogen species (ROS and RNS) are important signaling molecules that are involved in seed signaling pathways, cellular physiology, gene expression, differentiation, and growth [83]. Seed treatment with cold plasma and nanomaterials is an eco-agricultural high-tech technique that can increase crop yields [84,85]. The non-ionizing low-level radiation activates the vitality of a seed without causing gene mutation, so there is no genetic risk. A new path to increasing grain yield is provided by the application of cold plasma and nanotechnology in agriculture, since it is a fast, cheap, green, and riskless method [86]. Using cold plasma could help increase germination rates and peroxidase activities in seeds [87]. Figure 5 illustrates that both UV and biologically active agents (nitrogen and oxygen active species) produce molecular changes in seeds which then influence food composition [88]. In a study, Abedi et al. reported that seed priming with cold plasma enhanced early growth and flowering while protecting *Cichorium intybus* with selenium nanoparticles. This study provides a better understanding of the potential advantages of cold plasma in improving early growth, protection, and production. Figure 6 indicate that plasma with nano-selenium is capable of improving plant tolerance to stress conditions via activation of plant defense systems, especially antioxidants. As a result, plasma priming combined with nano-Selenium at a low dose is an effective approach to promote plant growth, biochemistry, and protection [89].

Using non-thermal plasma, A. Babajani et al. studied selenium and zinc oxide nanoparticle reactions of seed priming. Medicinal plant *Melissa officinalis* has shown to respond differently to zinc oxide (nZnO) and selenium (nSe) nanoparticles when primed with cold plasma. The primed seeds with plasma were cultured in nutrient solution modulated with nSe and nZnO. As a result of plasma priming, the growth-related characteristics (stem length, root length, and width of the leaf) as well as biomass accumulation were improved, as well as the toxicity signs of nSe were attenuated [88]. The study described differential physiology and expression of phenylalanine ammonia lyase (PAL) and universal stress protein (USP) in endangered species published by Moghanloo et al. (2019). Treatments with plasma and nano silicon (nSi) simultaneously had the highest expression rates of the gene for phenylalanine ammonia lyase. In contrast, the plasma treatments did not make a significant difference in the expression of USP gene, but nSi-treated seedlings exhibited higher levels of USP expression. After plasma and nSi were applied, leaf thickness and vascular tissue development were reinforced (xylem and phloem). In the present study, nSi and plasma as potential antagonists of phytotoxicity are described, which may be used as a theoretical base for possible commercialization [90]. Cold plasma has been reported to reduce the toxicity signs of nano zinc oxide in capsicum cayenne by modifying growth, differentiation, and physiology, according to A. Iranbakhsh et al. In this study, the systemically applied cold plasma solution and zinc oxide nanoparticles (nZnO) were evaluated within plant (*Capsicum annuum*) under in vitro and in pot conditions using a functional scientific device and metal-based nanoparticles. Eliciting peroxidase enzyme activity in both culture media was achieved by treating either with plasma or nZnO on both roots, and it was found that plasma activity and/or nZnO activity of phenylalanine ammonia-lyase were significantly greater. Plant growth was most influenced by soaking seeds with nanomaterials before plasma treatment in the pot experiment [91].

## 7. Artificial Intelligence and Machine Learning Technology for Nanoprimed Seed Diagnostics

**Figure 7:**
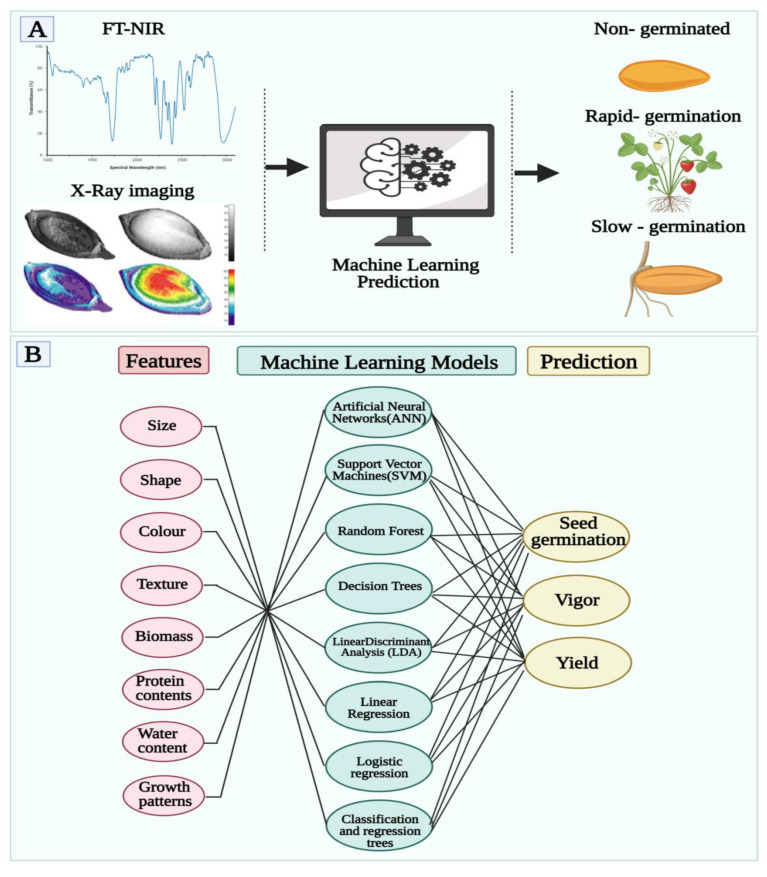
(**A**) Artificial intelligence and machine learning may assist with infrared spectroscopy and X-ray image data mining and curation. AI and ML may further complement via optimizing seed priming technology based on chemianalytic profiling for heat and moisture content assisting in breaking seed dormancy. (**B**) Post nanopriming, AI and ML tools can be used to predict seed germination, vigor, and yield of nanoprimed seeds.

Seeds with better seed germination and seedling emergence rates can ensure reliable emergence under a variety of agricultural conditions and are therefore instrumental in ensuring yield potential and uniformity. As of today, germination scoring is typically done by human observation, and therefore, has a limited frequency, scale, and accuracy. In order to handle this bottleneck, many attempts have been made to automate both seed diagnostics and associated phenotypic analysis, with more than one research-based solution such as germinator, phenoSeeder, and the MultiSense tool as a result [92]. As shown in Figure 7B, by using these softwares, post seed nanopriming diagnosis can be done to predict nanoprimed seeds viability and phenotypic analysis.

Recently, advanced computer vision and machine learning methods have been applied to germination assays; among them, the Rice Seed Germination Evaluation System which uses artificial neural networks to gauge the germination status of Thai rice species [93]. Numerous computer vision and machine learning that combined analytic methods have been developed to automate phenotypic analyses of organs as diverse as leaves, roots, and reproductive organs [94,95]. Nanoprimed seed germination can be measured dynamically and objectively based on the color, texture, morphology, and growth pattern of a seed, thus enabling new biological findings about seed physiology. In addition, the automation of seed germination scoring represents an excellent opportunity to start standardizing seed science research. The quality of seeds and their vigor can be accessed through digital analysis, but also biological experiments under varying conditions can be quantitatively compared to increase the confidence of research results. A study by Genze et al. presented accurate germination detection, prediction and quality assessment based on machine learning [96]. Several attempts have been made to automate seed testing in order to reduce the number of error-prone manual steps required in this process. Various analyses of seeds have recently been carried out using modern image analysis techniques since they are easily automatized and can provide unbiased and quantitative measurements which are error-free [97]. Modern convolutional networks were used in the present study to detect individual seeds and to distinguish germinated from non-germinated seeds with higher precision. By using artificial intelligence to measure germination potential, the evaluation process could be sped up. Compared to manual methods and conventional methods, it has a lower error rate and a higher performance. This allows it to provide more accurate germination indices and assess the quality of seeds. An approach for determining seed quality was developed using FT-NIR spectroscopy and X-ray imaging data by A D Medeiros et al. [98]. The results shown in Figure 7A illustrate how FT-NIR and X-ray imaging can be used in conjunction with machine learning algorithms to improve seed germination and vigor prediction. This study examines the use of FT-NIR spectroscopy in conjunction with X-ray imaging in the prediction of seed quality traits (germination and vigor). Seed germination capacity can be accurately measured (85% accuracy) using the proposed approach. A machine learning algorithm developed using both NIR spectra and X-ray images can easily, efficiently, and accurately predict seed germination. A D Medeiros et al. developed a system for classifying soybean seedlings based on quality data [98]. In this study, it was proposed to use traditional machine learning methods for determining the size, shape, and physiological potential of soybean seeds and seedlings. The models were developed using free and low-cost software based on images of soy seeds and seedlings. Using the developed model for seed and seedling classifications, 94% of seed and seedlings were accurately classified. By using interactive and traditional machine learning models, high precision was demonstrated in the models developed, which were able to classify soybean seeds by their appearance, as well as beans and their seedlings by vigor quickly and non-subjectively. A deep learning architecture is an artificial neural network (ANN) that is used to find patterns in data or model complex relationships between inputs and outputs. Classifiers are based on multilayer neural networks for identifying wheat grain (seed) irregularities grown in a variety of agricultural environmental zones. The scientific and technological developments of agricultural enterprises enable them to generate a large amount of data. Automated analysis methods are needed to process this data size. It is a very useful way to support agricultural center experts using advanced computing techniques [99,100]. Combined with real-time processing of data, it helps farmers take correct decisions concerning harvesting crops, planting, and fertilization. The following Table 2 displays some examples of seed analysis software with their features for possible use to diagnose nanoprimed seeds.

## 8. Limitations of Nanopriming Techniques and Future Prospects

It is recognized that despite remarkable progress, scientists do not have a complete understanding of how these nanomaterials can affect the macro- and micro-environments of seeds. It is alarming that there is still a lack of basic understanding regarding the possible health and safety effects of engineered nanomaterials on both human and non-human receptors considering the actual and projected levels of exposure [107]. A general rule concerning seed nanopriming does not exist, and there is no clear trend regarding priming responses depending on the taxonomic position of the species. As a result of some nanopriming treatments, there is a possibility of contamination of the medium with fungi and bacteria, which may greatly hinder subsequent seed germination [7]. A seed that has been nanoprimed is dried back to its original moisture content, but this process is done faster than the dehydration of mature seeds. Several researchers have hypothesized that brutal desiccation procedures alter the effects of nanopriming [7]. As a consequence, nanoprimed seed material can be less stable, and higher maintenance costs for seed companies and farmers are consequently incurred. In some cases, repeated nanopriming treatments can partly prevent seed viability losses, whereas in others, such losses are permanent and cannot be reversed [108]. Possibly requiring an additional treatment may be both an extra cost and a source of variability because germination potential may not be fully restored.

Nanopriming can be a potential future target for sustainable agriculture with the current global supply versus demand trend. A fortification program consists of adding vitamins and minerals to processed foods as a public health measure to improve the nutritional quality of the food supply. The goal is to enhance the nutrient intake by the population [52]. As a result, biofortification reduces the runoff of fertilizers and other agrochemicals and their inputs into the environment. As a result of seed biofortification, stands have been established and plant production, yields, nutrient content, and water utilization have increased as well as plant tolerance to biotic and abiotic stresses have increased [109]. The technique has been found to be a simple, practical, and cost-effective approach to improving the quality of seed and crops in resource-constrained regions. Compared to conventional seed priming, seed priming using engineered nanomaterial produced higher germination rates at equivalent or lower life cycle embodied energy. Nanotechnology is also an exceptionally potent way to deliver nutrients effectively due to its ability to deliver a wide range of engineered nanomaterials in a more efficient manner than before [34,110]. By introducing nano-bio-fortification into seeds, less fertilizer potentially may be consumed. This results in the need to use less water and less resources to cultivate the same volume of nutrient-rich food. Nano-bio fortification of seeds could also assist in shortening the growth cycle of plants (e.g., requiring less time for them to germinate and to mature), allowing the land to grow crops faster in the future. In similar ways to other agronomic methods, the success of seed nano-biofortification will depend on how well it is adapted to the setting, including the type of soil, the type of crop and the climate, and the location [111]. The advantages of nanotechnology need to be compared to other biofortification procedures that may provide long-term and cost-effective solutions, including plant breeding and CRISPR/transgenic technology [111]. In addition to assessing the overall environmental implications, it is important to determine if nano-based solutions are practical compared to conventional practices [112]. Nanomaterials continue to be manufactured in larger scales, which lead to further decreases in production costs. It is highly likely that nanotechnology will integrate with seed biofortification practices in the future, especially as highly specialized and tunable nanomaterials emerge. Nano-enabled seed biofortification is an important topic that merits intense scrutiny and greater attention given its potential benefits and the increasing global food insecurity that we will face in the coming decades.

## Figures and Tables

**Table 1:** An overview of nanoparticles used for seed priming, their physico-chemical properties, and the main effects on some species of seeds when evaluated against biotic and abiotic stress [7].

Type of Nanomaterials	Size of Nanomaterials	Concentrations of Nanomterials	Seed Species	Results	References
Chitosan nanoparticles containing zinc	387.7 ± 4 nm	0.01, 0.04, 0.08, 0.12, and 0.16% *w*/*v*	Maize seeds (*Zea mays* L.)	Improved seed and seedling vigor and biotic resistance	[38]
Chitosan nanoparticles containing copper	374.3 ± 8.2 nm	0.01, 0.04, 0.08, 0.12, and 0.16% *w*/*v*	Maize seeds (*Zea mays* L.)	Improved seed and seedling vigor	[39]
Chitosan loaded with gibberellic acid	450 ± 10 nm	0.05, 0.005, and 0.0005 mg/mL	Tomato (*Solanum lycopersicum* var. *cerasiforme*)	Improved seed vigor and plant morphology with increased biomass	[40]
Lignin nanoparticles loaded with gibberellic acid	200–250 nm	0.5, 1, and 1.5 mg/mL	Arugula (*Eruca visicaria* (L.) Cav. subsp. *sativa*), tomato (*Solanum lycopersicum* L. cv. Ciliegino), and chickpea (*Cicer arietinum* L.)	Improved seed and seedlings vigor	[41]
Cobalt and molybdenum oxide nanoparticles	60–80 nm	1 L/40 kg of seeds	Soybean seeds (*Glycine max* (L). Merr.)	Improved seed vigor and plant morphology with increased biomass	[42]
Multi-walled carbon nanotubes	13–14 nm	70, 80, and 90 µg/mL	Wheat (*Triticum aestivum* L.)	Improved seed vigor and plant morphology	[43]
Silver nanoparticles	141.3 ± 0.78 nm	31.3 µg/mL	Watermelon (*Citrullus lanatus* (Thunb.) Matsum. and Nakai)	Improved seed vigor and plant morphology	[44]
Iron nanoparticles	~80 nm	25, 50, 100, 200, 300, 400, 500, and 1000 µg/mL	Wheat (*Triticum aestivum* L.)	Improved seed vigor and plant morphology	[45]
Zinc nanoparticles	21.3 nm	20, 40, and 60 mg/L.	Lupin (*Lupinis termis* L.).	Increased salinity resistance and biochemical activity	[46]
Zinc nanoparticles	20 nm	1, 10, 100, 1000, and 5000 mg/L	Common bean (*Phaseolus vulgaris* L.)	Increased biomass	[47]
Copper nanoparticles	25, 40, and 80 nm	1, 10, 100, and 1000 mg/L	Common bean (*Phaseolus vulgaris* L.)	Increased seed vigor and biomass	[48]
Iron (II) sulfide aqua nanoparticles	6–20 nm	30 µg/mL	Rice (*Oryza sativa* L.)	Improved seed vigor and disease resistance	[49]
Manganese (III) oxide nanoparticles	50 nm	0.1, 0.5, and 1 mg/mL	Jalapeño (*Capsicum annuum* L.)	Increased salinity resistance and antioxidant enzymes	[50]
Chitosan/tripoly phosphate nanoparticles	259.4 ± 4.7 nm	1–100 µg/mL	Wheat (*Triticum aestivum* L.)	Improved plant morphologyand upregulation of plant growth regulator	[51]
Silicon nanoparticles	90 nm	300, 600, 900, and 1200 mg/L	Wheat (*Triticum aestivum* L.)	Increased biomass and biochemical activity, reduced cadmium uptake	[52]
Silver nanoparticles	6–26 nm	10 and 20 mg/mL	Rice seeds (*Oriza sativa* L. cv. KDML 105)	Upregulation of aquaporin gene expression, improved seed and seedlings vigor	[35]
Iron oxide nanoparticles	<50 nm	10, 50, 100, and 500 mg/L	Sorghum (*Sorghum bicolor* (L.) Moench)	Increased biochemical activity and biomass, improved water content in leaves	[53]
Iron nanoparticles	19–30 nm	20, 40, 80, and 160 mg/L	Watermelon (*Citrullus lanatus* (Thunb.) Matsum and Nakay varieties).	Improved plant morphology, reduced phytotoxicity	[54]
Zinc, titanium, and silver	ZnO, TiO2, A g 35–40, 100, 85 nm, resp.	750, 1000, and 1250 mg/kg	Chilli (*Capsicum annuum* L.)	Improved seed vigor, increased disease resistance	[55]

**Table 2:** Software that processes images simultaneously and produces accurate analyses of seedling germination and establishment. Using artificial neural networks, advanced computer-vision and machine-learning techniques have been applied to germination assays to assess the germination status of various nanoprimed seed.

Seed Analysis Software	Type of Seed Studied	Feature	Remark	Reference
Germinator	*Arabidopsis thaliana* seed	An indicator of germination	An automated high-throughput evaluation procedure for germination	[101]
Seed Vigor Imaging System	Soybean and Corn Seed	Used to calculate the length of seeds	Physiological differences between seed lots could be identified	[102]
SeedUSoon	*-*	Tracking of different mutations	It provides a visual representation of the genetic link between related seed batche	[103]
SmartGrain	*Arabidopsis thaliana* seed	Recognize varieties of seeds	Understanding the genes and mechanisms of grain or seed size and shape	[104]
SeedExtractor	Rice seeds	Measures seed size, shape (including length, width, circle and color	Crop yield traits can be determined	[105]
SeedCounter	Wheat grain	The use of a mobile device can be used to estimate the number and size of grains	It is possible to improve the accuracy of seed analysis.	[106]

## Data Availability

Data available in a publicly accessible repository.
